# (+)-*trans*-Chlorido­{2-[(*R*_p_)-2-(methyl­sulfan­yl)ferro­cen­yl]-2,5,6,7-tetra­hydro­pyrrolo­[1,2-*c*]imidazol-3-yl­idene}bis(tri­phenyl­phosphane-κ*P*)palladium(II) hexa­fluorido­phosphate di­chloro­form disolvate

**DOI:** 10.1107/S2056989016013190

**Published:** 2016-08-26

**Authors:** Cody Wilson-Konderka, Alan J. Lough, Costa Metallinos

**Affiliations:** a Department of Chemistry, Brock University, 1812 Sir Isaac Brock Way, St Catharines, ON, L2S 3A1, Canada; b Department of Chemistry, University of Toronto, 80 St George St., Toronto, ON, M5S 3H6, Canada

**Keywords:** crystal structure, *N*-heterocyclic carbene, palladium, thio­ether, planar chiral, pyrrolo­imidazolium

## Abstract

The title solvated complex, [FePd(C_5_H_5_)(C_12_H_13_N_2_S)Cl(C_18_H_15_P)_2_]PF_6_·2CHCl_3_, bearing a chiral ferrocenyl pyrrolo­imidazolyl­idene *N*-heterocyclic carbene (NHC) ligand, was synthesized by oxidative addition of a chloro­imidazolium salt to Pd(PPh_3_)_4_. The Pd^II^ ion is coordinated in a slightly distorted square-planar coordination geometry, with the Cl atom *trans* to the coordinating C atom of the pyrrolo­imidazolyl­idene ligand. The complex features a pendant thio­ether group that is not involved in coordination to Pd. In the crystal, weak C—H⋯F and C—H⋯π inter­actions connect the components of the structure, forming chains propagating along [1-10]. The fused pyrrolidine ring is in an envelope conformation, and the flap atom was refined as disordered over two sets of sites, with occupancies of 0.77 (4) and 0.23 (4).

## Abstract 2

A solvated palladium(II) complex bearing a planar chiral ferrocenyl pyrrolo­imidazolyl­idene (NHC) ligand, synthesized by oxidative addition of a chloro­imidazolium salt to Pd(PPh_3_)_4_, features a pendant thio­ether group that is not involved in coordination to Pd.

## Chemical context   

**Figure 1:**
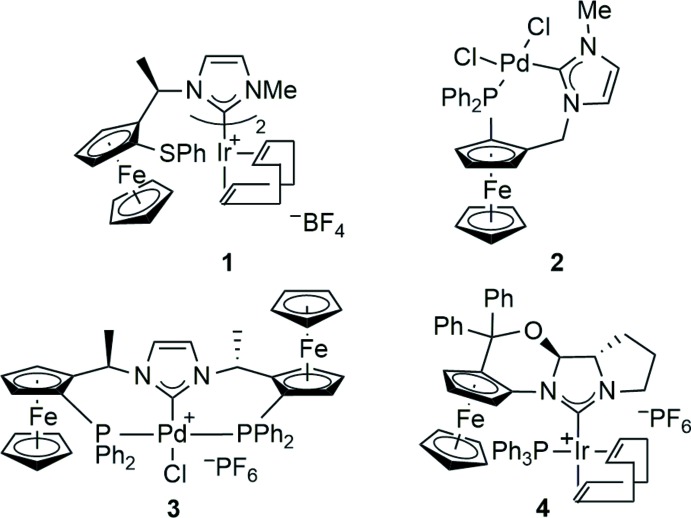
Coordination complexes with chiral ferrocenyl NHC ligands.

*N*-Heterocyclic carbenes (NHCs), such as imidazolylidenes, are electron-rich σ-donor ligands that may be electronically and sterically fine-tuned by changing the substituents on the azole ring (Clavier, 2006 ▸). These ligands exhibit weak π-back-bonding, resulting in increased electron density at the metal atom. Their overall electron-donating capacity is similar to that of tri­alkyl­phosphane ligands and is a main reason for inter­est in imidazolylidenes as ancillary ligands for transition-metal complexes with potential applications in catalysis (Hopkinson *et al.*, 2014 ▸). In general, higher electron density at transition metal atomshas been shown to promote oxidative addition steps in catalytic cycles (Peris, 2007 ▸). The selective synthesis of homochiral NHC ligands has been investigated concurrently with achiral forms. Particular attention has been paid to developing NHC ligands derived from planar chiral ferrocenes owing to the commercial importance of chiral ferrocene ligands, *e.g.* Josiphos (Schultz *et al.*, 2005 ▸), Xyliphos (Spindler *et al.*, 1990 ▸) and PhTRAP (Kuwano *et al.*, 2000 ▸). Some early examples of complexes bearing chiral ferrocenyl NHCs include Chung’s iridium complex **1**, in which the thio­ether group is not involved in metal ligation (Seo *et al.*, 2003 ▸) (Fig. 1 ▸). In contrast, bidentate **2** (Debono *et al.*, 2010 ▸) or tridentate pincer-like ferrocenyl NHC–phosphane ligands **3** (Gischig & Togni, 2004 ▸) have been prepared, which feature seven-membered palladacycles. Complex **2** has been shown to catalyze asymmetric Suzuki–Miyaura coupling of aryl bromides with naphthyl­boronic acids in up to 42% ee (Debono *et al.*, 2010 ▸). The preceding chiral ferrocenyl NHC ligands were prepared by initial diastereoselective li­thia­tion of Ugi’s amine (complexes **1** and **3**) (Marquarding *et al.*, 1970 ▸) or Kagan’s ferrocenyl acetal (complex **2**) (Riant *et al.*, 1993 ▸). We have recently reported that an iridium complex bearing a monodentate imidazolinyl­idene ligand catalyzes the hydrogenation of 2-substituted quinolines in up to 80% ee (John *et al.*, 2015 ▸). This ligand was prepared by diastereo­selective li­thia­tion of a ferrocene containing a new pyrrolo­imidazolone chiral auxiliary in which the N atom was directly attached to the cyclo­penta­dienyl (Cp) ring. The pyrrolo­imidazolone functionality doubled as a precursor to the NHC. In this sense, the NHC ligand in **4** is distinct from those in complexes **1–3**, which have ‘pendant’ imidazolylidenes. In this paper, we have extended this synthetic approach to prepare an unsaturated pyrrolo­imidazolyl­idene analogue of the ligands in complexes **1–3** to study its coordination behaviour with palladium. The crystal structure of the title compound, **8**, is presented herein.
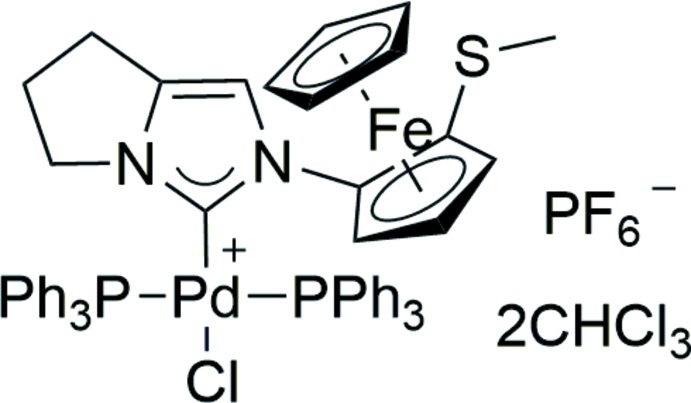


## Structural commentary   

**Figure 2:**
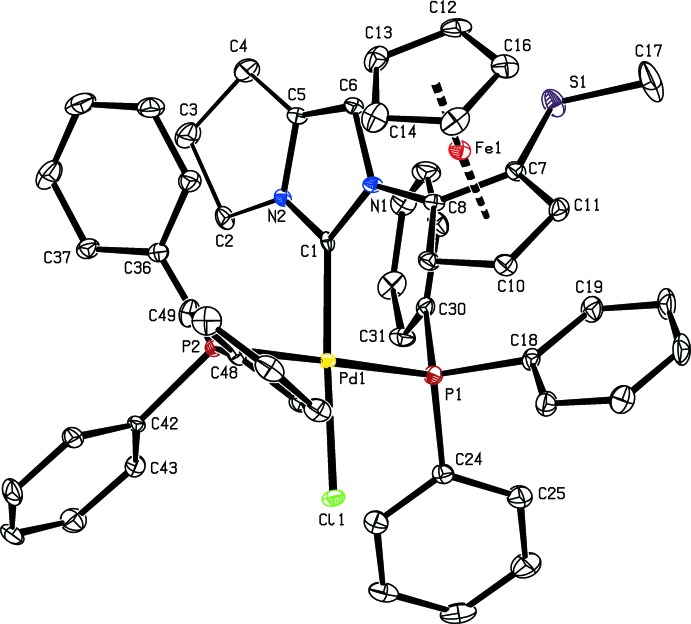
The mol­ecular structure of the cation of the title compound, shown with 30% probabilty displacement ellipsoids. H atoms have been omitted for clarity. The minor disorder component is not shown.

The mol­ecular structure of the title compound, **8**, is shown in Fig. 2 ▸. The Pd^II^ ion is coordinated in a slightly distorted square-planar coordination geometry, with the Cl atom *trans* to the pyrrolo­imidazolyl­idene ligand. The ligand is monodentate, with an *R*_p_ absolute configuration of the ferrocene moiety (Schlögl, 1967 ▸). The Schlögl convention has been used to assign planar chirality (*R*_p_ or *S*_p_) for consistency with our prior ferrocene work. As in iridium complex **1**, the thio­ether group is not involved in coordination to the metal atom in the title complex. The tri­phenyl­phosphane ligands are in slightly different chemical environments, an observation that is consistent with the non-equivalency of their P atoms by ^31^P NMR spectroscopy. The cyclo­penta­dienyl (Cp) rings of the ferrocenyl group are tilted slightly, by 2.75 (14)°, with respect to each other. The dihedral angle between the fused imidazole ring and the Cp ring to which it is attached is 46.1 (2)°. The fused pyrrolidine ring is in an envelope conformation, with atom C3 forming the flap. Atom C3 is disordered over two sites, with refined occupancies of 0.77 (4) and 0.23 (4). Within the cation, there are siginficant intra­molecular π–π stacking inter­actions, with centroid–centroid distances less than 4 Å namely, *Cg*1⋯*Cg*6 = 3.712 (3) Å, *Cg*2⋯*Cg*5 = 3.861 (8) Å, *Cg*2⋯*Cg*6 = 3.675 Å and *Cg*3⋯*Cg*4 = 3.641 Å, where *Cg*1, *Cg*2, *Cg*3, *Cg*4, *Cg*5 and *Cg*6 are the centroids of the N1/C1/N2/C4/C6, N2/C5/C4*A*/C3*A*/C2*A*, C7–C11, C18–C23, C30–C35 and C36–C41 rings, respectively.

## Supra­molecular features   

**Figure 3:**
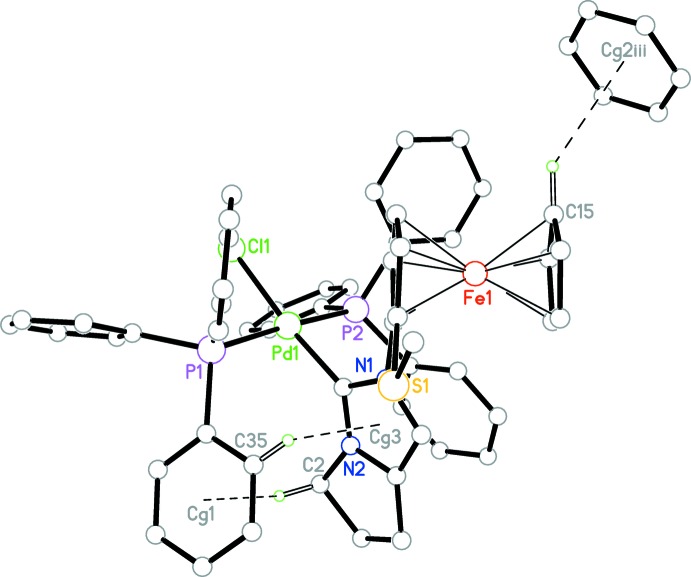
Part of the crystal structure of **8**, with weak C—H⋯π inter­actions shown as dashed lines. The centroids *Cg*1, *Cg*2 and *Cg*3, and the symmetry code are defined in Table 1 ▸. Only H atoms involved in weak inter­actions are shown.

In the crystal, weak C—H⋯F and C—H⋯π inter­actions connect the components of the structure, forming chains propagating along [1
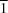
0] (Table 1 ▸, Figs. 3 ▸ and 4 ▸).

## Database survey   

A search of the Cambridge Structural Database (CSD, Version 5.37, update February 2015; Groom *et al.*, 2016 ▸) revealed only two structures where a Pd^II^ ion is coordinated to a tetra­hydro-1*H*-pyrrolo­[1,2-*c*]imidazol-3-yl­idene ligand, *viz.* *trans*-chloro­(2-phenyl-5,6,7,7a-tetra­hydro-1*H*-pyrrolo­[1,2-*c*]imidazol-3-yl­idene)bis­(tri­phenyl­phosphine)palladium(II) chloride di­chloro­methane solvate (CSD refcode XAMPOR; Kremzow *et al.*, 2005 ▸) and *trans*-chlorido­(2-phenyl-5,6,7,7a-tetra­hydro-1*H*-pyrrolo­[1,2-*c*]imidazol-3-yl­idene)bis­(tri­phenyl­phosphine)palladium(II) hexa­fluorido­phosphate di­chloro­methane solvate (XAMPIL; Kremzow *et al.*, 2005 ▸). The Pd—C_carbene_ bond length is 1.975 (2) and 1.9687 (17) Å in XAMPOR and XAMPIL, respectively, and these values are the same within experimental error as the value of 1.988 (5)Å in the title compound.

## Synthesis and crystallization   

### General   

**Figure 5:**
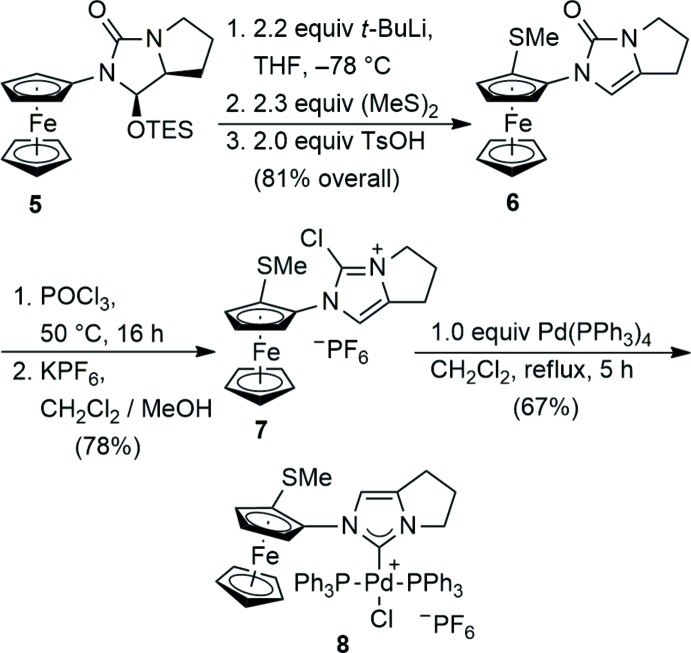
The reaction scheme.

The stereoselective synthesis of planar chiral ferrocene **6** by diastereoselective li­thia­tion has been reported previously (Metallinos *et al.*, 2012 ▸, 2013 ▸). Thus, sequential deprotonation of imidazolone **5**, followed by elecrophile quenching with dimethyl di­sulfide and subsequent acid-induced elimination of silanol, gave the chiral unsaturated urea **6**. Heating urea **6** in neat phospho­rus oxychloride in a sealed tube at 323 K resulted in the formation of chloro­imidazolium salt **7**, which was isolated as the hexa­fluorido­phosphate salt upon salt metathesis. Chloride **7** readily underwent oxidative addition with Pd(PPh_3_)_4_ according to the method of Fürstner *et al.* (2003 ▸) to give the title palladium complex **8** in 67% yield. Recrystallization of **8** from chloro­form solution containing a small amount of pentane gave the product as small yellow crystals that were suitable for X-ray diffraction. The reaction scheme is shown in Fig. 5 ▸.

### Preparation of (+)-3-chloro-2-[(*R*_p_)-2-(methyl­sulfan­yl)ferrocen­yl]-2,5,6,7-tetra­hydro­pyrrolo­[1,2-*c*]imidazol-4-ium hexa­fluoro­phosphate, 7   

A mixture of imidazolone **6** (147 mg, 0.42 mmol) in neat POCl_3_ (0.5 ml, 5.36 mmol) was heated at 323 K for 16 h. The resulting solution changed progressively from orange to black during this period. After cooling to room temperature, the volatiles were removed under high vacuum. The black residue obtained was dissolved in CH_2_Cl_2_ (10 ml) and treated with a saturated solution of KPF_6_ in H_2_O/MeOH (2 ml). The mixture was stirred for 15 min at room temperature, resulting in a colour change from black to deep red. Water was added (10 ml), resulting in a biphasic mixture from which the organic layer was isolated, washed with water, dried over anhydrous Na_2_SO_4_, filtered and concentrated under reduced pressure. The crude product was taken up in CH_2_Cl_2_ (2 ml) and added to an ice-cooled Et_2_O solution in an ice bath. The precipitate was collected by Hirsch funnel filtration and washed with cold Et_2_O to give a gold–beige powder [yield 161 mg, 78%; m.p. 368 K (Et_2_O)]. [α]_*D*_ +30.2 (*c* 1.0, CHCl_3_); IR (ATR, solid) ν_max_: 3152, 2977, 2923, 2875, 2858, 2851, 1650, 1537, 827 cm^−1^; ^1^H NMR (400 MHz, acetone-*d*_6_): δ 8.04 (*s*, 1H), 4.96 (*s*, 1H), 4.75 (*s*, 1H), 4.61 (*s*, 1H), 4.51 (*bs*, 7H), 3.23 (*s*, 2H), 2.80 (*s*, 2H), 2.21 (*s*, 3H); ^13^C NMR (100 MHz, CDCl_3_): δ 138.4, 128.1, 120.2, 93.9, 79.1, 72.3, 72.0, 68.1, 67.4, 48.6, 27.2, 24.0, 20.9; ESI–MS [*m*/*z* (%)]: 373 (M^+^, 100), 217 (5); HR–MS (ESI) calculated for C_17_H_18_ClFeN_2_S: 373.0229; found: 373.0222.

### Preparation of 8   

A solution of **7** (150 mg, 0.29 mmol) and Pd(PPh_3_)_4_ (334 mg, 0.13 mmol) in CH_2_Cl_2_ (25 ml) was heated under reflux for 5 h. After cooling, the solution was filtered through Celite, evaporated to dryness, and the crude product was recrystallized from CHCl_3_/pentane, to give bright-yellow powdery crystals [yield 246 mg, 67%; m.p. >503 K (CHCl_3_)]. [α]_*D*_ +25.1 (*c* 1.0, CHCl_3_); IR (ATR, solid) ν_max_: 3054, 1708, 1480, 1362 cm^−1^; ^1^H NMR (400 MHz, acetone-*d*_6_): δ 7.73 (*s*, 1H), 7.68–7.41 (*m*, 30H), 5.41 (*s*, 1H), 4.53 (*s*, 1H), 4.42 (*t*, 1H, *J* = 2.8 Hz), 4.17 (*s*, 5H), 3.18–3.12 (*m*, 1H), 3.03–2.97 (*m*, 1H), 2.36 (*t*, 2H, *J* = 7.2 Hz), 1.89 (*s*, 3H), 1.57 (quin, 2H, *J* = 7.6 Hz); ^13^C NMR (100 MHz, acetone-*d*_6_) δ 140.6, 134.2, 134.1, 131.7, 131.2, 129.2, 129.1, 128.7, 128.6, 120.5, 95.3, 79.0, 78.3, 71.3, 70.4, 66.1, 65.9, 46.8, 25.9, 22.3, 18.7; ^31^P NMR (162 MHz, acetone-*d*_6_): δ 30.1 (*s*, 1P), 20.6 (*s*, 1P), −144.5 [sept, 1P, ^1^ *J*(^31^P–^19^F) = 708 Hz]; ESI–MS [*m*/*z* (%)]: 1003 (36), 833 (100), 743 (35), 659 (24), 389 (66), 263 (41); HR–MS (ESI) calculated for C_53_H_48_N_2_ClFeP_2_PdS: 1003.1086; found: 1003.1126. Analysis calculated for C_53_H_48_N_2_ClF_6_FeP_3_PdS·CHCl_3_: C 55.37, H 4.21%; found: C 55.60, H 4.33%.

## Refinement   

Crystal data, data collection and structure refinement details are summarized in Table 2 ▸. H atoms were placed in calculated positions, with C—H = 0.95–1.00 Å, and included in a riding-model approximation, with *U*_iso_(H) = 1.5*U*_eq_(C) for methyl H atoms or 1.2*U*_eq_(C) otherwise. The flap atom, C3, of the fused pyrrolidine ring system was refined as disordered over two sites, with final occupancies of 0.77 (4) and 0.23 (4).

## Supplementary Material

Crystal structure: contains datablock(s) I. DOI: 10.1107/S2056989016013190/hb7606sup1.cif

Structure factors: contains datablock(s) I. DOI: 10.1107/S2056989016013190/hb7606Isup2.hkl

CCDC reference: 1499404

Additional supporting information: crystallographic information; 3D view; checkCIF report

## Figures and Tables

**Figure 4:**
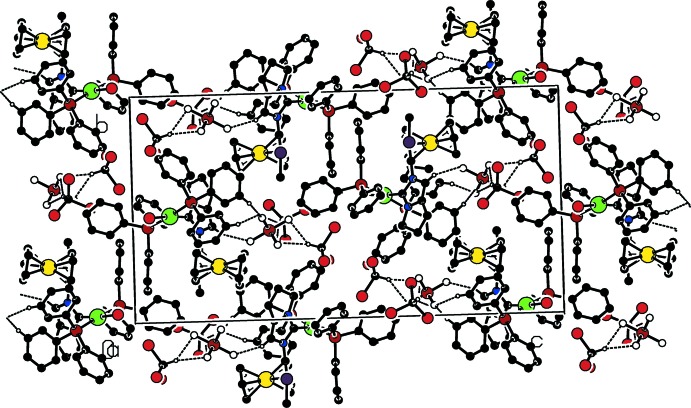
Part of the crystal structure of **8**, with weak C—H⋯F inter­actions shown as dashed lines. Only H atoms involved in weak inter­actions are shown.

**Table 1:** Hydrogen-bond geometry (Å, °) *Cg*1, *Cg*2 and *Cg*3 are the centroids of the C30–C35, C36–C41 and N1/C1/N2/C5/C6 rings, respectively.

*D*—H⋯*A*	*D*—H	H⋯*A*	*D*⋯*A*	*D*—H⋯*A*
C6—H6*A*⋯F3^i^	0.95	2.40	3.297 (6)	158
C40—H40*A*⋯F1^i^	0.95	2.52	3.327 (7)	143
C50—H50*A*⋯F4^ii^	0.95	2.38	3.275 (7)	156
C54—H54*A*⋯F4	1.00	2.42	3.342 (7)	153
C54—H54*A*⋯F6	1.00	2.33	3.237 (7)	150
C55—H55*A*⋯F5	1.00	2.44	3.228 (7)	135
C55—H55*A*⋯F6	1.00	2.33	3.311 (7)	168
C2—H2*B*⋯*Cg*1	0.99	2.88	3.682 (6)	139
C15—H15*A*⋯*Cg*2^iii^	1.00	2.93	3.762 (7)	141
C35—H35*A*⋯*Cg*3	0.95	2.67	3.148 (6)	111

Symmetry codes: (i) 
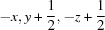
; (ii) 
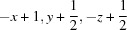
; (iii) 
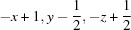
.

**Table 2:** Experimental details

Crystal data	
Chemical formula	[FePd(C_5_H_5_)(C_12_H_13_N_2_S)Cl(C_18_H_15_P)_2_]PF_6_·2CHCl_3_
*M*_r_	1388.34
Crystal system, space group	Orthorhombic, *P*2_1_2_1_2_1_
Temperature (K)	147
*a*, *b*, *c* (Å)	11.2517 (5), 16.424 (1), 31.1181 (18)
*V* (Å^3^)	5750.6 (5)
*Z*	4
Radiation type	Mo *K*α
μ (mm^−1^)	1.07
Crystal size (mm)	0.30 × 0.19 × 0.09

Data collection	
Diffractometer	Bruker Kappa APEX DUO CCD
Absorption correction	Multi-scan (*SADABS*; Bruker, 2014 ▸)
*T*_min_, *T*_max_	0.663, 0.746
No. of measured, independent and observed [*I* > 2σ(*I*)] reflections	30535, 13109, 10246
*R*_int_	0.058
(sin θ/λ)_max_ (Å^−1^)	0.650

Refinement	
*R*[*F* ^2^ > 2σ(*F* ^2^)], *wR*(*F* ^2^), *S*	0.044, 0.075, 0.98
No. of reflections	13109
No. of parameters	691
H-atom treatment	H-atom parameters constrained
Δρ_max_, Δρ_min_ (e Å^−3^)	0.53, −0.61
Absolute structure	Flack *x* determined using 3648 quotients [(*I* ^+^)−(*I* ^−^)]/[(*I* ^+^)+(*I* ^−^)] (Parsons *et al.*, 2013 ▸)
Absolute structure parameter	−0.011 (13)

Computer programs: *APEX2* and *SAINT* (Bruker, 2014 ▸), *SHELXT* (Sheldrick, 2015*a* ▸), *SHELXL2014* (Sheldrick, 2015*b* ▸), *PLATON* (Spek, 2009 ▸) and *SHELXTL* (Sheldrick, 2008 ▸).

## supplementary crystallographic information

### Crystal data

**Table:**

[FePd(C_5_H_5_)(C_12_H_13_N_2_S)Cl(C_18_H_15_P)_2_]PF_6_·2CHCl_3_	*D*_x_ = 1.604 Mg m^−^^3^
*M**_r_* = 1388.34	Mo *K*α radiation, λ = 0.71073 Å
Orthorhombic, *P*2_1_2_1_2_1_	Cell parameters from 5526 reflections
*a* = 11.2517 (5) Å	θ = 2.5–24.4°
*b* = 16.424 (1) Å	µ = 1.07 mm^−^^1^
*c* = 31.1181 (18) Å	*T* = 147 K
*V* = 5750.6 (5) Å^3^	Plate, orange
*Z* = 4	0.30 × 0.19 × 0.09 mm
*F*(000) = 2800	

### Data collection

**Table:**

Bruker Kappa APEX DUO CCD diffractometer	10246 reflections with *I* > 2σ(*I*)
Radiation source: sealed tube with Bruker Triumph monochromator	*R*_int_ = 0.058
φ and ω scans	θ_max_ = 27.5°, θ_min_ = 1.4°
Absorption correction: multi-scan (SADABS; Bruker, 2014)	*h* = −10→14
*T*_min_ = 0.663, *T*_max_ = 0.746	*k* = −21→20
30535 measured reflections	*l* = −40→40
13109 independent reflections	

### Refinement

**Table:**

Refinement on *F*^2^	Hydrogen site location: inferred from neighbouring sites
Least-squares matrix: full	H-atom parameters constrained
*R*[*F*^2^ > 2σ(*F*^2^)] = 0.044	*w* = 1/[σ^2^(*F*_o_^2^) + (0.0201*P*)^2^] where *P* = (*F*_o_^2^ + 2*F*_c_^2^)/3
*wR*(*F*^2^) = 0.075	(Δ/σ)_max_ = 0.001
*S* = 0.98	Δρ_max_ = 0.53 e Å^−^^3^
13109 reflections	Δρ_min_ = −0.61 e Å^−^^3^
691 parameters	Absolute structure: Flack *x* determined using 3648 quotients [(I+)-(I-)]/[(I+)+(I-)] (Parsons *et al.*, 2013)
0 restraints	Absolute structure parameter: −0.011 (13)

### Special details

**Table:**

Geometry. All e.s.d.'s (except the e.s.d. in the dihedral angle between two l.s. planes) are estimated using the full covariance matrix. The cell e.s.d.'s are taken into account individually in the estimation of e.s.d.'s in distances, angles and torsion angles; correlations between e.s.d.'s in cell parameters are only used when they are defined by crystal symmetry. An approximate (isotropic) treatment of cell e.s.d.'s is used for estimating e.s.d.'s involving l.s. planes.

### Fractional atomic coordinates and isotropic or equivalent isotropic displacement parameters (Å^2^)

**Table:**

	*x*	*y*	*z*	*U*_iso_*/*U*_eq_	Occ. (<1)
Pd1	0.33330 (3)	0.96585 (3)	0.41241 (2)	0.01254 (9)	
Fe1	0.27891 (7)	0.73959 (5)	0.30082 (3)	0.01854 (19)	
Cl1	0.49228 (11)	0.95869 (9)	0.46005 (4)	0.0200 (3)	
S1	0.00261 (13)	0.73694 (10)	0.34952 (5)	0.0287 (4)	
P1	0.19986 (12)	0.91810 (10)	0.46437 (5)	0.0162 (3)	
P2	0.46163 (11)	1.02430 (9)	0.36165 (4)	0.0139 (3)	
N1	0.1746 (4)	0.8971 (3)	0.34336 (13)	0.0138 (9)	
N2	0.1299 (3)	1.0203 (3)	0.35635 (13)	0.0132 (10)	
C1	0.2043 (4)	0.9615 (3)	0.36879 (15)	0.0118 (11)	
C2	0.1067 (5)	1.1032 (3)	0.37085 (19)	0.0197 (13)	0.77 (4)
H2A	0.1812	1.1351	0.3731	0.024*	0.77 (4)
H2B	0.0658	1.1036	0.3990	0.024*	0.77 (4)
C3	0.0267 (16)	1.1362 (6)	0.3354 (5)	0.034 (4)	0.77 (4)
H3A	0.0733	1.1701	0.3152	0.041*	0.77 (4)
H3B	−0.0374	1.1701	0.3478	0.041*	0.77 (4)
C4	−0.0263 (5)	1.0630 (4)	0.3119 (2)	0.0287 (16)	0.77 (4)
H4A	−0.1084	1.0518	0.3217	0.034*	0.77 (4)
H4B	−0.0268	1.0716	0.2804	0.034*	0.77 (4)
C2A	0.1067 (5)	1.1032 (3)	0.37085 (19)	0.0197 (13)	0.23 (4)
H2AA	0.1715	1.1405	0.3620	0.024*	0.23 (4)
H2AB	0.0980	1.1054	0.4025	0.024*	0.23 (4)
C3A	−0.011 (3)	1.125 (2)	0.3482 (13)	0.020 (9)*	0.23 (4)
H3AA	−0.0786	1.1220	0.3686	0.023*	0.23 (4)
H3AB	−0.0077	1.1813	0.3363	0.023*	0.23 (4)
C4A	−0.0263 (5)	1.0630 (4)	0.3119 (2)	0.0287 (16)	0.23 (4)
H4AA	−0.1095	1.0434	0.3103	0.034*	0.23 (4)
H4AB	−0.0037	1.0867	0.2838	0.034*	0.23 (4)
C5	0.0553 (5)	0.9959 (3)	0.32403 (18)	0.0168 (13)	
C6	0.0827 (5)	0.9181 (4)	0.31496 (18)	0.0181 (13)	
H6A	0.0472	0.8844	0.2937	0.022*	
C7	0.1584 (5)	0.7435 (3)	0.35010 (16)	0.0176 (12)	
C8	0.2245 (5)	0.8181 (3)	0.34678 (17)	0.0151 (12)	
C9	0.3460 (5)	0.7996 (3)	0.35239 (17)	0.0189 (13)	
H9A	0.4129	0.8396	0.3518	0.023*	
C10	0.3571 (5)	0.7146 (3)	0.35824 (18)	0.0211 (14)	
H10A	0.4333	0.6843	0.3623	0.025*	
C11	0.2422 (5)	0.6793 (4)	0.35640 (18)	0.0222 (13)	
H11A	0.2231	0.6201	0.3592	0.027*	
C12	0.1885 (6)	0.7210 (4)	0.24507 (18)	0.0298 (15)	
H12A	0.1001	0.7180	0.2422	0.036*	
C13	0.2576 (6)	0.7922 (4)	0.24119 (19)	0.0294 (16)	
H13A	0.2271	0.8483	0.2353	0.035*	
C14	0.3781 (6)	0.7700 (4)	0.24738 (19)	0.0319 (16)	
H14A	0.4480	0.8077	0.2468	0.038*	
C15	0.3817 (6)	0.6846 (4)	0.25522 (19)	0.0298 (16)	
H15A	0.4549	0.6517	0.2608	0.036*	
C16	0.2648 (5)	0.6545 (4)	0.25400 (18)	0.0258 (15)	
H16A	0.2402	0.5964	0.2579	0.031*	
C17	−0.0134 (6)	0.6283 (4)	0.3475 (3)	0.055 (2)	
H17A	−0.0972	0.6144	0.3431	0.082*	
H17B	0.0143	0.6046	0.3746	0.082*	
H17C	0.0341	0.6066	0.3237	0.082*	
C18	0.1928 (5)	0.8077 (3)	0.46366 (17)	0.0187 (13)	
C19	0.0873 (5)	0.7635 (4)	0.46357 (19)	0.0259 (14)	
H19A	0.0135	0.7914	0.4621	0.031*	
C20	0.0886 (6)	0.6799 (4)	0.4656 (2)	0.0335 (17)	
H20A	0.0163	0.6501	0.4649	0.040*	
C21	0.1954 (6)	0.6397 (4)	0.4685 (2)	0.0344 (17)	
H21A	0.1966	0.5820	0.4703	0.041*	
C22	0.3009 (5)	0.6824 (4)	0.4687 (2)	0.0301 (16)	
H22A	0.3742	0.6541	0.4710	0.036*	
C23	0.3002 (5)	0.7663 (4)	0.46567 (19)	0.0260 (14)	
H23A	0.3730	0.7956	0.4649	0.031*	
C24	0.2339 (5)	0.9427 (3)	0.52001 (17)	0.0185 (13)	
C25	0.1877 (5)	0.8926 (4)	0.55286 (19)	0.0293 (15)	
H25A	0.1502	0.8425	0.5458	0.035*	
C26	0.1973 (6)	0.9165 (5)	0.5950 (2)	0.0417 (18)	
H26A	0.1645	0.8833	0.6170	0.050*	
C27	0.2541 (6)	0.9886 (4)	0.6059 (2)	0.0377 (18)	
H27A	0.2600	1.0046	0.6352	0.045*	
C28	0.3021 (5)	1.0369 (4)	0.57395 (18)	0.0283 (14)	
H28A	0.3430	1.0855	0.5813	0.034*	
C29	0.2906 (5)	1.0145 (4)	0.53135 (19)	0.0236 (14)	
H29A	0.3221	1.0487	0.5095	0.028*	
C30	0.0494 (4)	0.9583 (4)	0.45903 (16)	0.0170 (12)	
C31	0.0073 (5)	1.0170 (4)	0.48745 (18)	0.0243 (14)	
H31A	0.0556	1.0338	0.5109	0.029*	
C32	−0.1039 (5)	1.0510 (4)	0.4820 (2)	0.0292 (16)	
H32A	−0.1317	1.0907	0.5018	0.035*	
C33	−0.1751 (5)	1.0276 (4)	0.44788 (19)	0.0293 (14)	
H33A	−0.2513	1.0513	0.4440	0.035*	
C34	−0.1343 (5)	0.9697 (4)	0.41967 (19)	0.0321 (15)	
H34A	−0.1825	0.9536	0.3961	0.039*	
C35	−0.0237 (5)	0.9346 (4)	0.42521 (18)	0.0222 (14)	
H35A	0.0026	0.8939	0.4057	0.027*	
C36	0.3880 (5)	1.0678 (4)	0.31492 (17)	0.0168 (13)	
C37	0.3671 (4)	1.1508 (4)	0.31238 (18)	0.0227 (14)	
H37A	0.3964	1.1858	0.3342	0.027*	
C38	0.3035 (5)	1.1832 (4)	0.2782 (2)	0.0351 (17)	
H38A	0.2898	1.2402	0.2767	0.042*	
C39	0.2600 (6)	1.1325 (5)	0.2462 (2)	0.0364 (17)	
H39A	0.2165	1.1546	0.2228	0.044*	
C40	0.2805 (5)	1.0496 (4)	0.24870 (19)	0.0304 (16)	
H40A	0.2516	1.0146	0.2267	0.036*	
C41	0.3427 (5)	1.0176 (4)	0.28306 (17)	0.0245 (14)	
H41A	0.3546	0.9604	0.2849	0.029*	
C42	0.5453 (5)	1.1094 (3)	0.38305 (17)	0.0159 (12)	
C43	0.5069 (5)	1.1491 (4)	0.41998 (18)	0.0227 (14)	
H43A	0.4395	1.1294	0.4351	0.027*	
C44	0.5664 (5)	1.2172 (4)	0.4348 (2)	0.0304 (16)	
H44A	0.5390	1.2442	0.4599	0.036*	
C45	0.6653 (5)	1.2462 (4)	0.4134 (2)	0.0314 (14)	
H45A	0.7071	1.2922	0.4241	0.038*	
C46	0.7027 (5)	1.2079 (4)	0.3764 (2)	0.0304 (16)	
H46A	0.7700	1.2280	0.3614	0.036*	
C47	0.6437 (5)	1.1410 (4)	0.36124 (18)	0.0220 (14)	
H47A	0.6699	1.1156	0.3355	0.026*	
C48	0.5722 (4)	0.9546 (4)	0.33932 (17)	0.0181 (13)	
C49	0.6155 (5)	0.9647 (4)	0.29755 (18)	0.0257 (13)	
H49A	0.5863	1.0077	0.2801	0.031*	
C50	0.7003 (5)	0.9124 (4)	0.2818 (2)	0.0320 (16)	
H50A	0.7295	0.9196	0.2534	0.038*	
C51	0.7431 (5)	0.8503 (4)	0.3064 (2)	0.0285 (15)	
H51A	0.8021	0.8148	0.2952	0.034*	
C52	0.7008 (5)	0.8388 (4)	0.3479 (2)	0.0259 (15)	
H52A	0.7301	0.7953	0.3650	0.031*	
C53	0.6160 (5)	0.8908 (4)	0.36398 (19)	0.0223 (14)	
H53A	0.5870	0.8829	0.3923	0.027*	
P3	0.04207 (14)	0.39566 (10)	0.31838 (5)	0.0232 (4)	
F1	−0.0651 (3)	0.4502 (2)	0.30243 (13)	0.0439 (10)	
F2	−0.0473 (3)	0.3301 (2)	0.33670 (13)	0.0457 (11)	
F3	0.0451 (3)	0.3494 (2)	0.27321 (12)	0.0430 (10)	
F4	0.1341 (3)	0.4618 (3)	0.30074 (12)	0.0460 (10)	
F5	0.0423 (3)	0.4412 (2)	0.36370 (11)	0.0403 (10)	
F6	0.1518 (3)	0.3408 (2)	0.33421 (12)	0.0435 (10)	
Cl2	0.4768 (3)	0.35412 (15)	0.35456 (11)	0.1039 (10)	
Cl3	0.3769 (2)	0.48557 (18)	0.40295 (7)	0.0862 (8)	
Cl4	0.4627 (2)	0.51171 (13)	0.31834 (6)	0.0663 (6)	
C54	0.3937 (6)	0.4421 (4)	0.3519 (2)	0.0364 (17)	
H54A	0.3133	0.4289	0.3401	0.044*	
Cl5	0.23776 (16)	0.25871 (15)	0.45352 (8)	0.0708 (7)	
Cl6	−0.01617 (14)	0.25246 (11)	0.44399 (6)	0.0420 (4)	
Cl7	0.09639 (14)	0.40164 (10)	0.47042 (5)	0.0331 (4)	
C55	0.1110 (5)	0.3136 (4)	0.4386 (2)	0.0331 (16)	
H55A	0.1190	0.3303	0.4078	0.040*	

### Atomic displacement parameters (Å^2^)

**Table:**

	*U*^11^	*U*^22^	*U*^33^	*U*^12^	*U*^13^	*U*^23^
Pd1	0.01091 (19)	0.0142 (2)	0.01251 (18)	−0.00038 (19)	−0.00128 (17)	0.00038 (18)
Fe1	0.0189 (4)	0.0177 (5)	0.0190 (4)	0.0020 (4)	−0.0011 (3)	−0.0042 (4)
Cl1	0.0177 (7)	0.0258 (8)	0.0165 (6)	0.0011 (7)	−0.0054 (5)	0.0020 (6)
S1	0.0185 (8)	0.0246 (9)	0.0429 (10)	−0.0025 (7)	0.0030 (7)	−0.0020 (8)
P1	0.0159 (8)	0.0190 (9)	0.0138 (7)	−0.0016 (6)	0.0015 (6)	0.0000 (6)
P2	0.0132 (7)	0.0133 (8)	0.0151 (7)	0.0003 (6)	−0.0004 (5)	0.0003 (6)
N1	0.011 (2)	0.014 (2)	0.017 (2)	0.004 (2)	−0.004 (2)	−0.0015 (18)
N2	0.015 (2)	0.010 (3)	0.014 (2)	0.0006 (19)	−0.0003 (17)	−0.0033 (19)
C1	0.015 (3)	0.007 (3)	0.013 (2)	−0.001 (2)	0.0049 (19)	−0.003 (2)
C2	0.022 (3)	0.010 (3)	0.028 (3)	0.001 (3)	0.000 (3)	−0.004 (2)
C3	0.042 (8)	0.023 (6)	0.038 (7)	0.008 (5)	−0.012 (6)	0.005 (5)
C4	0.024 (3)	0.024 (4)	0.038 (4)	0.008 (3)	−0.010 (3)	0.004 (3)
C2A	0.022 (3)	0.010 (3)	0.028 (3)	0.001 (3)	0.000 (3)	−0.004 (2)
C4A	0.024 (3)	0.024 (4)	0.038 (4)	0.008 (3)	−0.010 (3)	0.004 (3)
C5	0.014 (3)	0.019 (3)	0.017 (3)	−0.001 (2)	−0.003 (2)	−0.001 (2)
C6	0.015 (3)	0.021 (3)	0.018 (3)	−0.004 (3)	−0.004 (2)	0.002 (3)
C7	0.017 (3)	0.016 (3)	0.021 (3)	0.000 (3)	−0.002 (2)	−0.002 (2)
C8	0.014 (3)	0.014 (3)	0.017 (3)	0.005 (2)	−0.003 (2)	−0.005 (2)
C9	0.020 (3)	0.017 (3)	0.021 (3)	0.000 (3)	−0.004 (3)	−0.003 (2)
C10	0.015 (3)	0.020 (3)	0.028 (3)	0.005 (2)	−0.002 (2)	−0.002 (3)
C11	0.027 (3)	0.015 (3)	0.024 (3)	0.003 (3)	−0.001 (3)	0.001 (2)
C12	0.035 (4)	0.037 (4)	0.018 (3)	0.004 (3)	−0.009 (3)	−0.009 (3)
C13	0.044 (4)	0.026 (4)	0.018 (3)	0.003 (3)	0.003 (3)	0.000 (3)
C14	0.032 (4)	0.038 (4)	0.025 (4)	0.003 (3)	0.013 (3)	0.001 (3)
C15	0.034 (4)	0.031 (4)	0.025 (4)	0.015 (3)	0.005 (3)	−0.003 (3)
C16	0.031 (4)	0.024 (4)	0.022 (3)	0.006 (3)	0.001 (3)	−0.012 (3)
C17	0.032 (4)	0.028 (4)	0.105 (7)	−0.013 (3)	0.001 (4)	0.006 (4)
C18	0.026 (4)	0.017 (3)	0.013 (3)	−0.002 (3)	0.004 (2)	0.001 (2)
C19	0.023 (3)	0.030 (4)	0.025 (3)	−0.004 (3)	0.007 (3)	0.005 (3)
C20	0.041 (4)	0.027 (4)	0.032 (4)	−0.013 (3)	0.010 (3)	0.008 (3)
C21	0.051 (5)	0.014 (4)	0.038 (4)	−0.006 (3)	0.004 (3)	0.004 (3)
C22	0.030 (4)	0.026 (4)	0.034 (4)	0.007 (3)	0.003 (3)	0.002 (3)
C23	0.025 (3)	0.022 (4)	0.030 (3)	0.000 (3)	0.000 (3)	0.004 (3)
C24	0.013 (3)	0.023 (4)	0.019 (3)	0.000 (2)	0.001 (2)	0.000 (2)
C25	0.034 (4)	0.033 (4)	0.021 (3)	−0.006 (3)	−0.002 (3)	−0.001 (3)
C26	0.052 (5)	0.055 (5)	0.018 (4)	−0.010 (4)	−0.001 (3)	0.004 (3)
C27	0.039 (4)	0.055 (5)	0.019 (3)	0.009 (3)	−0.008 (3)	−0.009 (3)
C28	0.024 (3)	0.031 (4)	0.029 (3)	0.004 (3)	−0.008 (2)	−0.011 (3)
C29	0.018 (3)	0.027 (4)	0.026 (3)	0.001 (3)	0.002 (2)	−0.001 (3)
C30	0.014 (3)	0.020 (3)	0.017 (3)	−0.001 (3)	0.002 (2)	0.004 (3)
C31	0.018 (3)	0.033 (4)	0.022 (3)	−0.002 (3)	−0.004 (2)	−0.002 (3)
C32	0.023 (3)	0.035 (4)	0.030 (4)	0.011 (3)	0.007 (3)	0.001 (3)
C33	0.012 (3)	0.039 (4)	0.037 (3)	0.003 (3)	0.003 (3)	0.009 (3)
C34	0.019 (3)	0.047 (4)	0.031 (4)	−0.006 (3)	−0.006 (2)	0.004 (4)
C35	0.016 (3)	0.026 (4)	0.025 (3)	−0.006 (3)	0.008 (2)	−0.001 (3)
C36	0.013 (3)	0.023 (4)	0.014 (3)	−0.001 (2)	0.002 (2)	0.001 (2)
C37	0.018 (3)	0.028 (4)	0.022 (3)	0.001 (3)	−0.003 (2)	0.004 (3)
C38	0.034 (4)	0.036 (4)	0.036 (4)	0.009 (3)	0.000 (3)	0.014 (3)
C39	0.030 (4)	0.052 (5)	0.027 (4)	0.003 (3)	−0.009 (3)	0.010 (3)
C40	0.032 (4)	0.040 (5)	0.019 (3)	−0.005 (3)	−0.006 (3)	−0.001 (3)
C41	0.023 (3)	0.029 (4)	0.021 (3)	−0.004 (3)	−0.001 (3)	0.003 (3)
C42	0.015 (3)	0.011 (3)	0.021 (3)	0.000 (2)	−0.007 (2)	0.000 (2)
C43	0.028 (3)	0.020 (3)	0.020 (3)	−0.002 (3)	−0.001 (3)	0.002 (3)
C44	0.040 (4)	0.025 (4)	0.026 (4)	0.002 (3)	−0.004 (3)	−0.006 (3)
C45	0.031 (3)	0.022 (3)	0.042 (4)	−0.009 (3)	−0.018 (4)	−0.001 (3)
C46	0.018 (3)	0.026 (4)	0.047 (4)	−0.008 (3)	−0.002 (3)	0.009 (3)
C47	0.018 (3)	0.026 (4)	0.022 (3)	−0.005 (3)	−0.001 (3)	0.002 (3)
C48	0.013 (3)	0.017 (3)	0.024 (3)	−0.003 (2)	0.001 (2)	−0.002 (3)
C49	0.027 (3)	0.024 (3)	0.026 (3)	0.001 (3)	0.008 (2)	0.005 (3)
C50	0.026 (4)	0.041 (4)	0.029 (4)	0.004 (3)	0.010 (3)	−0.004 (3)
C51	0.022 (3)	0.029 (4)	0.035 (4)	0.003 (3)	0.003 (3)	−0.014 (3)
C52	0.018 (3)	0.024 (4)	0.036 (4)	0.007 (3)	−0.004 (3)	−0.006 (3)
C53	0.017 (3)	0.029 (4)	0.021 (3)	−0.005 (3)	−0.004 (2)	0.001 (3)
P3	0.0225 (8)	0.0243 (10)	0.0228 (8)	0.0017 (7)	−0.0005 (7)	−0.0012 (7)
F1	0.035 (2)	0.039 (3)	0.057 (3)	0.0086 (18)	−0.0135 (19)	0.004 (2)
F2	0.044 (2)	0.032 (2)	0.061 (3)	−0.0056 (19)	0.012 (2)	0.009 (2)
F3	0.056 (3)	0.045 (3)	0.028 (2)	0.000 (2)	−0.0096 (18)	−0.0111 (19)
F4	0.041 (2)	0.047 (3)	0.050 (2)	−0.013 (2)	0.0207 (18)	−0.002 (2)
F5	0.055 (2)	0.039 (3)	0.027 (2)	0.0074 (19)	0.0021 (18)	−0.0083 (17)
F6	0.038 (2)	0.046 (3)	0.046 (2)	0.019 (2)	−0.0145 (19)	−0.011 (2)
Cl2	0.114 (2)	0.0344 (14)	0.163 (3)	0.0246 (14)	−0.023 (2)	0.0040 (17)
Cl3	0.0986 (18)	0.119 (2)	0.0407 (13)	0.0073 (16)	0.0180 (11)	−0.0052 (13)
Cl4	0.0958 (16)	0.0643 (16)	0.0387 (11)	−0.0165 (13)	−0.0048 (11)	0.0144 (10)
C54	0.033 (4)	0.032 (4)	0.044 (4)	0.002 (3)	−0.007 (3)	−0.001 (3)
Cl5	0.0399 (11)	0.0659 (16)	0.107 (2)	0.0233 (11)	−0.0198 (12)	−0.0372 (14)
Cl6	0.0407 (10)	0.0397 (11)	0.0455 (11)	−0.0087 (9)	−0.0010 (8)	−0.0128 (9)
Cl7	0.0367 (9)	0.0280 (10)	0.0346 (10)	−0.0003 (8)	−0.0027 (8)	−0.0059 (7)
C55	0.034 (4)	0.041 (4)	0.025 (4)	0.000 (3)	0.001 (3)	−0.010 (3)

### Geometric parameters (Å, º)

**Table:**

Pd1—C1	1.988 (5)	C20—H20A	0.9500
Pd1—Cl1	2.3261 (13)	C21—C22	1.379 (8)
Pd1—P1	2.3416 (15)	C21—H21A	0.9500
Pd1—P2	2.3454 (14)	C22—C23	1.381 (8)
Fe1—C8	2.020 (5)	C22—H22A	0.9500
Fe1—C16	2.025 (6)	C23—H23A	0.9500
Fe1—C9	2.029 (5)	C24—C29	1.386 (8)
Fe1—C10	2.033 (6)	C24—C25	1.412 (8)
Fe1—C12	2.034 (6)	C25—C26	1.372 (8)
Fe1—C11	2.035 (6)	C25—H25A	0.9500
Fe1—C15	2.041 (6)	C26—C27	1.387 (9)
Fe1—C7	2.048 (5)	C26—H26A	0.9500
Fe1—C13	2.061 (6)	C27—C28	1.382 (8)
Fe1—C14	2.064 (6)	C27—H27A	0.9500
S1—C7	1.757 (6)	C28—C29	1.381 (8)
S1—C17	1.795 (7)	C28—H28A	0.9500
P1—C18	1.814 (6)	C29—H29A	0.9500
P1—C24	1.819 (6)	C30—C31	1.391 (8)
P1—C30	1.825 (5)	C30—C35	1.391 (7)
P2—C42	1.813 (5)	C31—C32	1.380 (7)
P2—C36	1.819 (6)	C31—H31A	0.9500
P2—C48	1.828 (6)	C32—C33	1.386 (8)
N1—C1	1.362 (6)	C32—H32A	0.9500
N1—C6	1.403 (6)	C33—C34	1.372 (9)
N1—C8	1.419 (6)	C33—H33A	0.9500
N2—C1	1.335 (6)	C34—C35	1.382 (8)
N2—C5	1.369 (6)	C34—H34A	0.9500
N2—C2	1.459 (7)	C35—H35A	0.9500
N2—C2A	1.459 (7)	C36—C41	1.386 (7)
C2—C3	1.524 (10)	C36—C37	1.387 (8)
C2—H2A	0.9900	C37—C38	1.387 (8)
C2—H2B	0.9900	C37—H37A	0.9500
C3—C4	1.529 (12)	C38—C39	1.387 (9)
C3—H3A	0.9900	C38—H38A	0.9500
C3—H3B	0.9900	C39—C40	1.384 (9)
C4—C5	1.483 (8)	C39—H39A	0.9500
C4—H4A	0.9900	C40—C41	1.382 (8)
C4—H4B	0.9900	C40—H40A	0.9500
C2A—C3A	1.55 (3)	C41—H41A	0.9500
C2A—H2AA	0.9900	C42—C43	1.390 (7)
C2A—H2AB	0.9900	C42—C47	1.398 (7)
C3A—C4A	1.53 (3)	C43—C44	1.382 (8)
C3A—H3AA	0.9900	C43—H43A	0.9500
C3A—H3AB	0.9900	C44—C45	1.381 (8)
C4A—C5	1.483 (8)	C44—H44A	0.9500
C4A—H4AA	0.9900	C45—C46	1.379 (9)
C4A—H4AB	0.9900	C45—H45A	0.9500
C5—C6	1.345 (7)	C46—C47	1.367 (8)
C6—H6A	0.9500	C46—H46A	0.9500
C7—C11	1.427 (8)	C47—H47A	0.9500
C7—C8	1.436 (7)	C48—C53	1.390 (8)
C8—C9	1.412 (7)	C48—C49	1.398 (7)
C9—C10	1.413 (7)	C49—C50	1.374 (8)
C9—H9A	1.0000	C49—H49A	0.9500
C10—C11	1.418 (8)	C50—C51	1.364 (8)
C10—H10A	1.0000	C50—H50A	0.9500
C11—H11A	1.0000	C51—C52	1.388 (8)
C12—C13	1.409 (9)	C51—H51A	0.9500
C12—C16	1.418 (8)	C52—C53	1.375 (8)
C12—H12A	1.0000	C52—H52A	0.9500
C13—C14	1.418 (8)	C53—H53A	0.9500
C13—H13A	1.0000	P3—F2	1.579 (4)
C14—C15	1.425 (9)	P3—F1	1.583 (4)
C14—H14A	1.0000	P3—F5	1.596 (4)
C15—C16	1.406 (8)	P3—F3	1.598 (4)
C15—H15A	1.0000	P3—F4	1.599 (4)
C16—H16A	1.0000	P3—F6	1.606 (4)
C17—H17A	0.9800	Cl2—C54	1.723 (7)
C17—H17B	0.9800	Cl3—C54	1.751 (7)
C17—H17C	0.9800	Cl4—C54	1.733 (7)
C18—C23	1.389 (7)	C54—H54A	1.0000
C18—C19	1.391 (8)	Cl5—C55	1.750 (6)
C19—C20	1.375 (8)	Cl6—C55	1.756 (7)
C19—H19A	0.9500	Cl7—C55	1.760 (6)
C20—C21	1.373 (9)	C55—H55A	1.0000
			
C1—Pd1—Cl1	173.96 (16)	Fe1—C13—H13A	126.2
C1—Pd1—P1	89.50 (14)	C13—C14—C15	107.7 (6)
Cl1—Pd1—P1	92.08 (5)	C13—C14—Fe1	69.8 (3)
C1—Pd1—P2	90.25 (14)	C15—C14—Fe1	68.8 (4)
Cl1—Pd1—P2	88.65 (5)	C13—C14—H14A	126.2
P1—Pd1—P2	175.39 (6)	C15—C14—H14A	126.2
C8—Fe1—C16	157.8 (2)	Fe1—C14—H14A	126.2
C8—Fe1—C9	40.8 (2)	C16—C15—C14	108.4 (5)
C16—Fe1—C9	159.0 (2)	C16—C15—Fe1	69.2 (3)
C8—Fe1—C10	68.8 (2)	C14—C15—Fe1	70.6 (3)
C16—Fe1—C10	121.8 (2)	C16—C15—H15A	125.8
C9—Fe1—C10	40.7 (2)	C14—C15—H15A	125.8
C8—Fe1—C12	123.2 (2)	Fe1—C15—H15A	125.8
C16—Fe1—C12	40.9 (2)	C15—C16—C12	107.5 (6)
C9—Fe1—C12	159.0 (2)	C15—C16—Fe1	70.4 (3)
C10—Fe1—C12	159.3 (3)	C12—C16—Fe1	69.9 (3)
C8—Fe1—C11	69.3 (2)	C15—C16—H16A	126.2
C16—Fe1—C11	105.1 (3)	C12—C16—H16A	126.2
C9—Fe1—C11	68.9 (2)	Fe1—C16—H16A	126.2
C10—Fe1—C11	40.8 (2)	S1—C17—H17A	109.5
C12—Fe1—C11	123.4 (3)	S1—C17—H17B	109.5
C8—Fe1—C15	161.1 (2)	H17A—C17—H17B	109.5
C16—Fe1—C15	40.4 (2)	S1—C17—H17C	109.5
C9—Fe1—C15	123.6 (2)	H17A—C17—H17C	109.5
C10—Fe1—C15	106.1 (2)	H17B—C17—H17C	109.5
C12—Fe1—C15	67.9 (3)	C23—C18—C19	119.1 (5)
C11—Fe1—C15	119.4 (3)	C23—C18—P1	116.8 (4)
C8—Fe1—C7	41.3 (2)	C19—C18—P1	124.0 (4)
C16—Fe1—C7	120.6 (2)	C20—C19—C18	120.8 (6)
C9—Fe1—C7	68.8 (2)	C20—C19—H19A	119.6
C10—Fe1—C7	68.6 (2)	C18—C19—H19A	119.6
C12—Fe1—C7	108.2 (2)	C21—C20—C19	119.5 (6)
C11—Fe1—C7	40.9 (2)	C21—C20—H20A	120.3
C15—Fe1—C7	155.4 (2)	C19—C20—H20A	120.3
C8—Fe1—C13	109.5 (2)	C20—C21—C22	120.6 (6)
C16—Fe1—C13	68.5 (3)	C20—C21—H21A	119.7
C9—Fe1—C13	123.5 (2)	C22—C21—H21A	119.7
C10—Fe1—C13	157.8 (2)	C21—C22—C23	120.1 (6)
C12—Fe1—C13	40.3 (2)	C21—C22—H22A	119.9
C11—Fe1—C13	161.0 (2)	C23—C22—H22A	119.9
C15—Fe1—C13	68.0 (3)	C22—C23—C18	119.8 (5)
C7—Fe1—C13	125.7 (2)	C22—C23—H23A	120.1
C8—Fe1—C14	125.4 (3)	C18—C23—H23A	120.1
C16—Fe1—C14	68.3 (3)	C29—C24—C25	118.8 (5)
C9—Fe1—C14	108.6 (3)	C29—C24—P1	121.9 (4)
C10—Fe1—C14	121.5 (2)	C25—C24—P1	118.8 (4)
C12—Fe1—C14	67.7 (3)	C26—C25—C24	119.7 (6)
C11—Fe1—C14	155.8 (2)	C26—C25—H25A	120.2
C15—Fe1—C14	40.6 (2)	C24—C25—H25A	120.2
C7—Fe1—C14	162.5 (2)	C25—C26—C27	121.0 (6)
C13—Fe1—C14	40.2 (2)	C25—C26—H26A	119.5
C7—S1—C17	99.3 (3)	C27—C26—H26A	119.5
C18—P1—C24	104.1 (3)	C28—C27—C26	119.5 (6)
C18—P1—C30	108.6 (3)	C28—C27—H27A	120.2
C24—P1—C30	101.6 (2)	C26—C27—H27A	120.2
C18—P1—Pd1	110.76 (18)	C29—C28—C27	120.1 (6)
C24—P1—Pd1	116.59 (18)	C29—C28—H28A	120.0
C30—P1—Pd1	114.26 (18)	C27—C28—H28A	120.0
C42—P2—C36	103.2 (3)	C28—C29—C24	120.9 (6)
C42—P2—C48	105.6 (2)	C28—C29—H29A	119.5
C36—P2—C48	104.6 (3)	C24—C29—H29A	119.5
C42—P2—Pd1	112.85 (19)	C31—C30—C35	118.3 (5)
C36—P2—Pd1	114.75 (18)	C31—C30—P1	120.6 (4)
C48—P2—Pd1	114.77 (19)	C35—C30—P1	121.1 (4)
C1—N1—C6	110.8 (4)	C32—C31—C30	120.7 (5)
C1—N1—C8	124.7 (4)	C32—C31—H31A	119.6
C6—N1—C8	124.3 (4)	C30—C31—H31A	119.6
C1—N2—C5	112.7 (4)	C31—C32—C33	120.3 (6)
C1—N2—C2	134.2 (4)	C31—C32—H32A	119.8
C5—N2—C2	113.0 (4)	C33—C32—H32A	119.8
C1—N2—C2A	134.2 (4)	C34—C33—C32	119.3 (5)
C5—N2—C2A	113.0 (4)	C34—C33—H33A	120.3
N2—C1—N1	103.8 (4)	C32—C33—H33A	120.3
N2—C1—Pd1	129.0 (4)	C33—C34—C35	120.7 (6)
N1—C1—Pd1	127.1 (4)	C33—C34—H34A	119.7
N2—C2—C3	102.3 (5)	C35—C34—H34A	119.7
N2—C2—H2A	111.3	C34—C35—C30	120.7 (6)
C3—C2—H2A	111.3	C34—C35—H35A	119.7
N2—C2—H2B	111.3	C30—C35—H35A	119.7
C3—C2—H2B	111.3	C41—C36—C37	118.8 (5)
H2A—C2—H2B	109.2	C41—C36—P2	120.4 (4)
C2—C3—C4	107.3 (7)	C37—C36—P2	120.6 (4)
C2—C3—H3A	110.3	C36—C37—C38	120.6 (6)
C4—C3—H3A	110.3	C36—C37—H37A	119.7
C2—C3—H3B	110.3	C38—C37—H37A	119.7
C4—C3—H3B	110.3	C39—C38—C37	120.1 (6)
H3A—C3—H3B	108.5	C39—C38—H38A	119.9
C5—C4—C3	102.7 (6)	C37—C38—H38A	119.9
C5—C4—H4A	111.2	C40—C39—C38	119.5 (6)
C3—C4—H4A	111.2	C40—C39—H39A	120.3
C5—C4—H4B	111.2	C38—C39—H39A	120.3
C3—C4—H4B	111.2	C41—C40—C39	120.1 (6)
H4A—C4—H4B	109.1	C41—C40—H40A	119.9
N2—C2A—C3A	103.4 (14)	C39—C40—H40A	119.9
N2—C2A—H2AA	111.1	C40—C41—C36	120.9 (6)
C3A—C2A—H2AA	111.1	C40—C41—H41A	119.6
N2—C2A—H2AB	111.1	C36—C41—H41A	119.6
C3A—C2A—H2AB	111.1	C43—C42—C47	118.3 (5)
H2AA—C2A—H2AB	109.0	C43—C42—P2	120.3 (4)
C4A—C3A—C2A	106 (2)	C47—C42—P2	121.3 (4)
C4A—C3A—H3AA	110.6	C44—C43—C42	120.3 (6)
C2A—C3A—H3AA	110.6	C44—C43—H43A	119.8
C4A—C3A—H3AB	110.6	C42—C43—H43A	119.8
C2A—C3A—H3AB	110.6	C45—C44—C43	120.5 (6)
H3AA—C3A—H3AB	108.7	C45—C44—H44A	119.7
C5—C4A—C3A	103.9 (13)	C43—C44—H44A	119.7
C5—C4A—H4AA	111.0	C46—C45—C44	119.4 (6)
C3A—C4A—H4AA	111.0	C46—C45—H45A	120.3
C5—C4A—H4AB	111.0	C44—C45—H45A	120.3
C3A—C4A—H4AB	111.0	C47—C46—C45	120.5 (6)
H4AA—C4A—H4AB	109.0	C47—C46—H46A	119.7
C6—C5—N2	106.9 (5)	C45—C46—H46A	119.7
C6—C5—C4	142.6 (5)	C46—C47—C42	120.9 (6)
N2—C5—C4	110.5 (5)	C46—C47—H47A	119.5
C6—C5—C4A	142.6 (5)	C42—C47—H47A	119.5
N2—C5—C4A	110.5 (5)	C53—C48—C49	118.7 (5)
C5—C6—N1	105.7 (5)	C53—C48—P2	120.3 (4)
C5—C6—H6A	127.2	C49—C48—P2	121.1 (5)
N1—C6—H6A	127.2	C50—C49—C48	119.9 (6)
C11—C7—C8	107.3 (5)	C50—C49—H49A	120.0
C11—C7—S1	127.9 (5)	C48—C49—H49A	120.0
C8—C7—S1	124.6 (4)	C51—C50—C49	120.9 (6)
C11—C7—Fe1	69.1 (3)	C51—C50—H50A	119.5
C8—C7—Fe1	68.3 (3)	C49—C50—H50A	119.5
S1—C7—Fe1	130.6 (3)	C50—C51—C52	120.1 (6)
C9—C8—N1	126.1 (5)	C50—C51—H51A	119.9
C9—C8—C7	108.0 (5)	C52—C51—H51A	119.9
N1—C8—C7	125.6 (5)	C53—C52—C51	119.5 (6)
C9—C8—Fe1	69.9 (3)	C53—C52—H52A	120.2
N1—C8—Fe1	130.6 (4)	C51—C52—H52A	120.2
C7—C8—Fe1	70.4 (3)	C52—C53—C48	120.9 (6)
C8—C9—C10	108.3 (5)	C52—C53—H53A	119.6
C8—C9—Fe1	69.3 (3)	C48—C53—H53A	119.6
C10—C9—Fe1	69.8 (3)	F2—P3—F1	90.8 (2)
C8—C9—H9A	125.9	F2—P3—F5	90.1 (2)
C10—C9—H9A	125.9	F1—P3—F5	90.8 (2)
Fe1—C9—H9A	125.9	F2—P3—F3	90.4 (2)
C9—C10—C11	108.5 (5)	F1—P3—F3	90.6 (2)
C9—C10—Fe1	69.5 (3)	F5—P3—F3	178.6 (2)
C11—C10—Fe1	69.7 (3)	F2—P3—F4	178.8 (2)
C9—C10—H10A	125.7	F1—P3—F4	90.1 (2)
C11—C10—H10A	125.7	F5—P3—F4	89.1 (2)
Fe1—C10—H10A	125.7	F3—P3—F4	90.4 (2)
C10—C11—C7	107.8 (5)	F2—P3—F6	89.8 (2)
C10—C11—Fe1	69.5 (3)	F1—P3—F6	179.4 (3)
C7—C11—Fe1	70.0 (3)	F5—P3—F6	89.4 (2)
C10—C11—H11A	126.1	F3—P3—F6	89.2 (2)
C7—C11—H11A	126.1	F4—P3—F6	89.3 (2)
Fe1—C11—H11A	126.1	Cl2—C54—Cl4	109.8 (4)
C13—C12—C16	108.8 (6)	Cl2—C54—Cl3	110.9 (4)
C13—C12—Fe1	70.9 (3)	Cl4—C54—Cl3	109.0 (4)
C16—C12—Fe1	69.2 (3)	Cl2—C54—H54A	109.0
C13—C12—H12A	125.6	Cl4—C54—H54A	109.0
C16—C12—H12A	125.6	Cl3—C54—H54A	109.0
Fe1—C12—H12A	125.6	Cl5—C55—Cl6	110.1 (4)
C12—C13—C14	107.7 (6)	Cl5—C55—Cl7	110.5 (3)
C12—C13—Fe1	68.8 (3)	Cl6—C55—Cl7	109.9 (3)
C14—C13—Fe1	70.0 (4)	Cl5—C55—H55A	108.8
C12—C13—H13A	126.2	Cl6—C55—H55A	108.8
C14—C13—H13A	126.2	Cl7—C55—H55A	108.8
			
C5—N2—C1—N1	−0.4 (6)	C30—P1—C18—C23	177.5 (4)
C2—N2—C1—N1	176.1 (5)	Pd1—P1—C18—C23	51.2 (5)
C2A—N2—C1—N1	176.1 (5)	C24—P1—C18—C19	101.6 (5)
C5—N2—C1—Pd1	177.4 (4)	C30—P1—C18—C19	−6.1 (6)
C2—N2—C1—Pd1	−6.0 (8)	Pd1—P1—C18—C19	−132.3 (4)
C2A—N2—C1—Pd1	−6.0 (8)	C23—C18—C19—C20	0.2 (9)
C6—N1—C1—N2	0.9 (6)	P1—C18—C19—C20	−176.2 (5)
C8—N1—C1—N2	−175.5 (4)	C18—C19—C20—C21	1.2 (10)
C6—N1—C1—Pd1	−177.0 (4)	C19—C20—C21—C22	−1.0 (10)
C8—N1—C1—Pd1	6.6 (7)	C20—C21—C22—C23	−0.6 (10)
C1—N2—C2—C3	169.7 (10)	C21—C22—C23—C18	2.0 (10)
C5—N2—C2—C3	−13.8 (10)	C19—C18—C23—C22	−1.8 (9)
N2—C2—C3—C4	20.0 (15)	P1—C18—C23—C22	174.8 (5)
C2—C3—C4—C5	−19.1 (15)	C18—P1—C24—C29	154.7 (5)
C1—N2—C2A—C3A	−166.8 (18)	C30—P1—C24—C29	−92.5 (5)
C5—N2—C2A—C3A	9.7 (19)	Pd1—P1—C24—C29	32.4 (5)
N2—C2A—C3A—C4A	−17 (3)	C18—P1—C24—C25	−33.7 (5)
C2A—C3A—C4A—C5	18 (3)	C30—P1—C24—C25	79.2 (5)
C1—N2—C5—C6	−0.3 (6)	Pd1—P1—C24—C25	−156.0 (4)
C2—N2—C5—C6	−177.6 (5)	C29—C24—C25—C26	1.5 (9)
C2A—N2—C5—C6	−177.6 (5)	P1—C24—C25—C26	−170.4 (5)
C1—N2—C5—C4	179.2 (5)	C24—C25—C26—C27	−1.5 (10)
C2—N2—C5—C4	1.9 (6)	C25—C26—C27—C28	−0.2 (10)
C1—N2—C5—C4A	179.2 (5)	C26—C27—C28—C29	1.7 (9)
C2A—N2—C5—C4A	1.9 (6)	C27—C28—C29—C24	−1.6 (9)
C3—C4—C5—C6	−170.0 (11)	C25—C24—C29—C28	0.0 (8)
C3—C4—C5—N2	10.9 (11)	P1—C24—C29—C28	171.7 (4)
C3A—C4A—C5—C6	166 (2)	C18—P1—C30—C31	129.8 (5)
C3A—C4A—C5—N2	−12.7 (19)	C24—P1—C30—C31	20.5 (5)
N2—C5—C6—N1	0.8 (6)	Pd1—P1—C30—C31	−106.0 (4)
C4—C5—C6—N1	−178.3 (7)	C18—P1—C30—C35	−53.8 (5)
C4A—C5—C6—N1	−178.3 (7)	C24—P1—C30—C35	−163.1 (5)
C1—N1—C6—C5	−1.1 (6)	Pd1—P1—C30—C35	70.4 (5)
C8—N1—C6—C5	175.3 (5)	C35—C30—C31—C32	−0.3 (9)
C17—S1—C7—C11	−11.5 (6)	P1—C30—C31—C32	176.2 (4)
C17—S1—C7—C8	172.7 (5)	C30—C31—C32—C33	−0.5 (9)
C17—S1—C7—Fe1	82.6 (5)	C31—C32—C33—C34	0.5 (9)
C1—N1—C8—C9	−43.9 (8)	C32—C33—C34—C35	0.3 (9)
C6—N1—C8—C9	140.2 (6)	C33—C34—C35—C30	−1.2 (9)
C1—N1—C8—C7	128.4 (6)	C31—C30—C35—C34	1.2 (8)
C6—N1—C8—C7	−47.5 (8)	P1—C30—C35—C34	−175.3 (4)
C1—N1—C8—Fe1	−137.7 (5)	C42—P2—C36—C41	161.6 (5)
C6—N1—C8—Fe1	46.4 (7)	C48—P2—C36—C41	51.4 (5)
C11—C7—C8—C9	−1.8 (6)	Pd1—P2—C36—C41	−75.2 (5)
S1—C7—C8—C9	174.7 (4)	C42—P2—C36—C37	−24.1 (5)
Fe1—C7—C8—C9	−60.0 (4)	C48—P2—C36—C37	−134.3 (4)
C11—C7—C8—N1	−175.3 (5)	Pd1—P2—C36—C37	99.1 (4)
S1—C7—C8—N1	1.2 (8)	C41—C36—C37—C38	−1.0 (8)
Fe1—C7—C8—N1	126.5 (5)	P2—C36—C37—C38	−175.4 (4)
C11—C7—C8—Fe1	58.2 (4)	C36—C37—C38—C39	0.2 (9)
S1—C7—C8—Fe1	−125.2 (4)	C37—C38—C39—C40	0.0 (10)
N1—C8—C9—C10	174.7 (5)	C38—C39—C40—C41	0.7 (10)
C7—C8—C9—C10	1.2 (6)	C39—C40—C41—C36	−1.6 (9)
Fe1—C8—C9—C10	−59.1 (4)	C37—C36—C41—C40	1.7 (8)
N1—C8—C9—Fe1	−126.2 (5)	P2—C36—C41—C40	176.1 (4)
C7—C8—C9—Fe1	60.3 (4)	C36—P2—C42—C43	105.2 (5)
C8—C9—C10—C11	−0.1 (7)	C48—P2—C42—C43	−145.3 (4)
Fe1—C9—C10—C11	−58.9 (4)	Pd1—P2—C42—C43	−19.2 (5)
C8—C9—C10—Fe1	58.8 (4)	C36—P2—C42—C47	−69.5 (5)
C9—C10—C11—C7	−1.0 (7)	C48—P2—C42—C47	40.0 (5)
Fe1—C10—C11—C7	−59.8 (4)	Pd1—P2—C42—C47	166.1 (4)
C9—C10—C11—Fe1	58.8 (4)	C47—C42—C43—C44	−1.1 (8)
C8—C7—C11—C10	1.8 (6)	P2—C42—C43—C44	−175.9 (4)
S1—C7—C11—C10	−174.6 (4)	C42—C43—C44—C45	−0.6 (9)
Fe1—C7—C11—C10	59.5 (4)	C43—C44—C45—C46	1.6 (9)
C8—C7—C11—Fe1	−57.7 (4)	C44—C45—C46—C47	−0.9 (9)
S1—C7—C11—Fe1	125.9 (4)	C45—C46—C47—C42	−0.8 (9)
C16—C12—C13—C14	0.5 (7)	C43—C42—C47—C46	1.8 (8)
Fe1—C12—C13—C14	59.5 (4)	P2—C42—C47—C46	176.6 (4)
C16—C12—C13—Fe1	−59.1 (4)	C42—P2—C48—C53	94.1 (5)
C12—C13—C14—C15	−0.2 (7)	C36—P2—C48—C53	−157.4 (4)
Fe1—C13—C14—C15	58.6 (4)	Pd1—P2—C48—C53	−30.8 (5)
C12—C13—C14—Fe1	−58.8 (4)	C42—P2—C48—C49	−85.2 (5)
C13—C14—C15—C16	−0.2 (7)	C36—P2—C48—C49	23.3 (5)
Fe1—C14—C15—C16	59.0 (4)	Pd1—P2—C48—C49	149.9 (4)
C13—C14—C15—Fe1	−59.2 (4)	C53—C48—C49—C50	−0.5 (8)
C14—C15—C16—C12	0.4 (7)	P2—C48—C49—C50	178.8 (5)
Fe1—C15—C16—C12	60.3 (4)	C48—C49—C50—C51	0.1 (9)
C14—C15—C16—Fe1	−59.9 (4)	C49—C50—C51—C52	0.3 (9)
C13—C12—C16—C15	−0.6 (7)	C50—C51—C52—C53	−0.4 (9)
Fe1—C12—C16—C15	−60.6 (4)	C51—C52—C53—C48	0.0 (8)
C13—C12—C16—Fe1	60.1 (4)	C49—C48—C53—C52	0.5 (8)
C24—P1—C18—C23	−74.8 (5)	P2—C48—C53—C52	−178.8 (4)

### Hydrogen-bond geometry (Å, º)

*Cg*1, *Cg*2 abd *Cg*3 are the centroids of the C30–C35, C36–C41 and N1/C1/N2/C5/C6 rings, respectively.

**Table:**

*D*—H···*A*	*D*—H	H···*A*	*D*···*A*	*D*—H···*A*
C6—H6*A*···F3^i^	0.95	2.40	3.297 (6)	158
C40—H40*A*···F1^i^	0.95	2.52	3.327 (7)	143
C50—H50*A*···F4^ii^	0.95	2.38	3.275 (7)	156
C54—H54*A*···F4	1.00	2.42	3.342 (7)	153
C54—H54*A*···F6	1.00	2.33	3.237 (7)	150
C55—H55*A*···F5	1.00	2.44	3.228 (7)	135
C55—H55*A*···F6	1.00	2.33	3.311 (7)	168
C2—H2*B*···*Cg*1	0.99	2.88	3.682 (6)	139
C15—H15*A*···*Cg*2^iii^	1.00	2.93	3.762 (7)	141
C35—H35*A*···*Cg*3	0.95	2.67	3.148 (6)	111

Symmetry codes: (i) −*x*, *y*+1/2, −*z*+1/2; (ii) −*x*+1, *y*+1/2, −*z*+1/2; (iii) −*x*+1, *y*−1/2, −*z*+1/2.
